# Glycoprotein composition along the pistil of *Malus* x *domestica* and the modulation of pollen tube growth

**DOI:** 10.1186/1471-2229-14-1

**Published:** 2014-01-03

**Authors:** Juan M Losada, Maria Herrero

**Affiliations:** 1 Pomology Department, Aula Dei Experimental Station CSIC, Apdo 13034, 50080 Zaragoza, Spain; 2 Present address: Arnold Arboretum of Harvard University, 1300 Centre Street, 02131 Boston, MA, USA

**Keywords:** Arabinogalactan proteins, AGPs, Apple, Callose, Extensins, EXTs, *Malus* x *domestica*, Pistil, Pollen tube, Style, Transmitting tissue

## Abstract

**Background:**

The characteristics of pollen tube growth are not constant, but display distinct patterns of growth within the different tissues of the pistil. In the stigma, the growth rate is slow and autotrophic, whereas in the style, it is rapid and heterotrophic. Very little is known about the interactions between these distinct maternal tissues and the traversing pollen tube and the role of this interaction on the observed metabolism. In this work we characterise pollen tube growth in the apple flower and look for differences in glycoprotein epitope localization between two different maternal tissues, the stigma and the style.

**Results:**

While immunocytochemically-detected arabinogalactan proteins were present at high levels in the stigma, they were not detected in the transmitting tissue of the style, where extensins were abundant. Whereas extensins remained at high levels in unpollinated pistils, they were no longer present in the style following pollen tube passage. Similarily, while abundant in unpollinated styles, insoluble polysaccharides such as β-glucans, were depleted in pollinated pistils.

**Conclusions:**

The switch from autotropic to heterotrophic pollen tube growth correlates spatially with a change of glycoprotein epitopes between the stigma and the style. The depletion of extensins and polysaccharides following pollen tube passage in the style suggest a possible contribution to the acceleration of heterotrophic pollen tube growth, which would imply an active contribution of female tissues on prezygotic male–female crosstalk.

## Background

The development and function of the male gametophyte is amazingly well conserved across flowering plants. Following pollen grain hydration, a pollen tube starts to develop. The vegetative cell, hosting the generative cell or the two sperm cells, travels along extensible tip of the pollen tube, the area where the new wall is built. This continuous construction of a new wall at the apical end allows the growth of the pollen tube tip to be oriented towards the female gametophyte [1]. This highly conserved process, however, contrasts with clear differences found in the speed at which this wall is built up. Comparison of the process in multiple species shows that evolutionary derived flowering seed plants have a faster pollen tube growth rate than extant non-flowering seed plants, as well as early divergent angiosperms [2,3].

Pollen tube growth is complex and highly variable depending on the conditions. Since early work in the 1940s, it has been shown that the pollen tube growth rate is dependent on temperature in a wide range of species [4-6]. The growth rate in *in vitro* germination media is lower compared to *in vivo* conditions [7]. Additionally, the pollen tube growth rate also varies depending on the surrounding maternal tissue. The pollen tube traverses different tissues within the pistil on its path to the embryo sac, and the recorded pollen tube growth rate is faster in the style compared to the stigma or the ovary [8,9]. As observed in different species such as peaches [10] or alders [11], slow growth in the ovary has been related to stops and decelerations resulting from the wait for particular structures -the obturator or the ovules- to become receptive to the pollen tube. Indeed, male–female synchrony appears to be a prerequisite for successful fertilization [12].

The requirement for coordinated timing between gametophytes raises the question of why pollen tubes cross large styles at high speeds. Darwin (1886) [13], puzzled on how pollen tubes rapidly covered long styles, suggested that some kind of support was provided by the style. In the 1970s, the incorporation of labelled compounds from the style into growing pollen tubes [14], together with the fact that starch was depleted from the style as pollen tubes passed by [15], suggested that a change from an autotrophic to heterotrophic pollen tube growth in the style was associated with an acceleration in pollen tube growth rate [8,16].

In the centre of the style, either a canal or a transmitting tract composed of specialized secretory tissues through which pollen tubes undergo tip oriented growth, represents the location for male–female molecular interactions [17,18]. In species with hollow styles, such as *Lilium*, the secretory cells bordering the stylar canal exert a crucial role by providing adhesion and chemotropic guidance cues for the growing pollen tubes [19,20]. Some of these cues also appear to play a part in species with solid styles [21]. The stylar transmitting tissue of most dicots is composed of elongated cells [22] that secrete a pectinaceous extracellular matrix. In *Arabidopsis*, the non-proteic amino acid γ-aminobutiric acid (GABA) is needed for pollen tube elongation in the ovary [23]. In mutants lacking GABA, the expression of genes encoding secreted proteins of the cell wall in the ovarian transmitting tissue is also affected [24]. However, since the pollen tube wall is mainly carbohydrate based, a nutritional relationship likely relies on individual polysaccharides or glycoconjugates such as glycoproteins.

Hydroxyproline-rich glycoproteins (HPRGPs), a superfamily of abundant glycoproteins in styles, have been also detected in pollen tube walls in abundant levels [25,26].Within this superfamily, arabinogalactan proteins (AGPs) are involved in a number of developmental processes during the plant life cycle [27]. AGPs have been found in the style of *Nicotiana*; in particular, the transmitting tissue-specific glycoprotein (TTS) has been shown to attract and stimulate pollen tubes following a deglycosylation gradient [28,29] as well as providing guidance cues for growing pollen tubes [30,31]. Additionally, extensins (EXTs), another group of HPRGPs normally related to cell elongation processes [32,33], actively participate in the progamic phase in some species. EXTs have been reported in reproductive tissues of some angiosperms, for example the pistil specific extensin-like protein (PELPIII) of Solanaceae was recently shown to play a role in interspecific incompatibility in *Nicotiana*[34]. PELPIII also shows biochemical characteristics of AGPs and prevent interspecific hybridization [35]. Putting all this information together indicates that the style supports pollen tube growth [36], and that a modulator effect on pollen tube wall formation occurs due to an intense provision of nutrients and signals from the female tissues to the male pollen tubes [37,38]. Exogenous application of specific compounds can mimic the role of the style to support pollen tube growth in mutants with a hollow style [39].

On the male side, expansion of the pollen tube wall occurs at a fast rate and contains mostly de-esterified pectins and callose at their shank and esterified pectins and cellulose at the proximal growing region [40]. During pollen tube elongation, callose plugs isolate the active tube tip from the rest of the tube [41]. Extensin-like proteins become detectable during pollen tube elongation [42]. In fact, the best characterised extensins are the non-classical, pollen specific extensin-like proteins (Pex) from maize and tomato, which have been localized to the inner callose layer of pollen tubes [43]. These extensins contain the leucine rich repeat domain (LRR), a motif shared between dicots and monocots, and are putatively involved in communication [44]. Thus far, however, the role for “classic” extensins during pollen-pistil interactions remains elusive.

This still fragmented information is difficult to put together by the fact that the genetic control of maternal tissues over pollen tubes is elusive, apart from stylar incompatibility mutations, no pistil mutants have been reported that arrest pollen tube growth in the style during compatible matings, even though TTS glycoproteins play a clear part in pollen tube growth [29,30]. As an alternative, immunocytology can shed light on this process by identifying the proteins present in the pistil and their spatial distribution following pollination. This approach has been successfully used in the stigma of the apple flower; the detection of two particular AGP epitopes (via JIM 8 and JIM 13), is associated with the acquisition of stigmatic receptivity [45]. The disappearance of these AGP epitopes is correlated with pollen tube growth. Information is still lacking on the relationship between glycoprotein detection and how the style provides provisions for pollen tube wall building. In this work, we follow pollen tube growth rate in the apple style and evaluate the pistil support of pollen tube wall formation. It is known that polysaccharides [46] and S-RNases are present in the transmitting tissue cells of apple [47]. Our results illustrate the localization differences between glycoprotein epitopes in the intercellular spaces of the stigma and the style. While β-glucans and extensins are present in abundant levels in unpollinated pistils, they decrease in abundance and become undetectable in the style as the pollen tubes pass by.

## Results

### Pollen tube growth rates and transmitting tissue characterization

**Figure 1:**
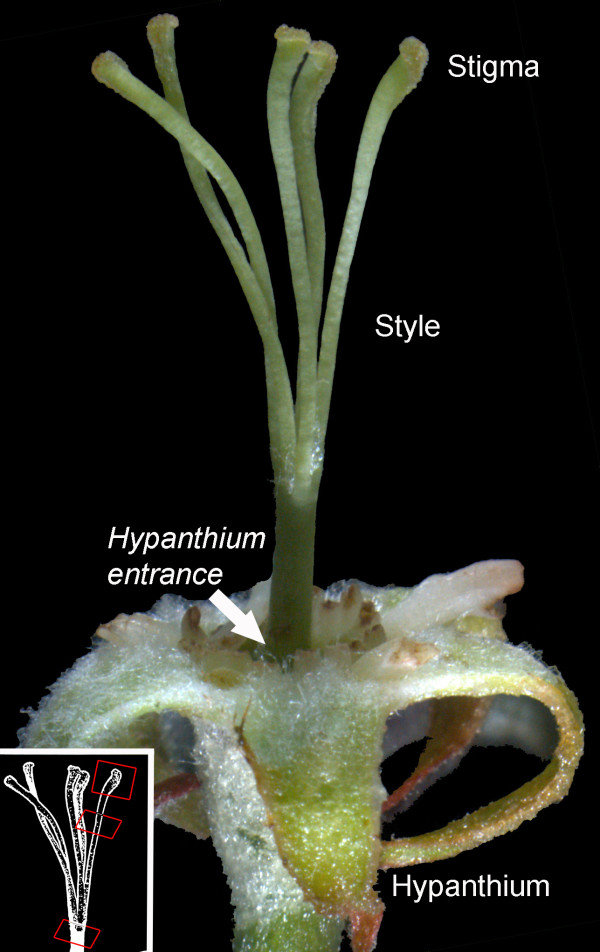
Pentacarpelar apple pistil showing individual stylar parts from top (stigma) to bottom (hypanthium), and scheme of the sections along the style (insert, red squares) evaluated in this work.

**Figure 2:**
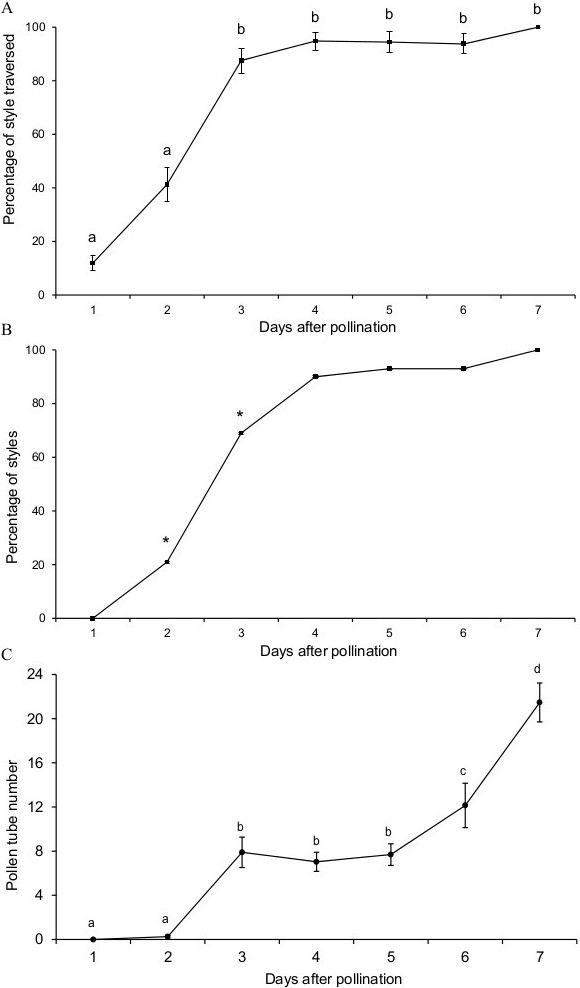
**Pollen tube growth rate in the apple style. (A)** Percentage of the style traversed by the longest pollen tube. **(B)** Percentage of styles with pollen tubes at their base. **(C)** Mean number of pollen tubes at the style base. Letters over points are independent significant groups separated by Duncan multiple range test at a p?≤?0.05. Asterisks represent significant dependence between day after pollination and the percentage of style traversed with chi-square homogeneity test at a p?≤?0.05.

Apple pentacarpelar pistils have five individual styles. In apple as in other Pomoideae, the base of the styles and the ovaries are enclosed within a tissue, the hypanthium, which at maturity constitutes the edible part of the fruit (Figure 1). On the stigmatic surface, pollen grains germinated by two hours after pollination and grew along the stigma surface. By ten hours after pollination, pollen tubes penetrated the transmitting tissue in the area of the suture line, covering a distance of some 700 μm from the edge of the stigma to the suture line. This results in a pollen tube growth rate of 87 μm?·?h^-1^ [700 μm/(10 h-2 h)] in the stigma. Once in the style, the longest pollen tube had travelled 50% of the length of the style two days after pollination and 90% of the length of the style three days after pollination (Figure 2A). In the style, pollen tubes grew 11 mm in 62 hours (72 h – 10 h for stigma growth), equalling a growth rate of roughly 177 μm?·?h^-1^. Pollen tube growth varied between styles, and the hypanthium entrance was reached in 20% of the styles two days after pollination, and in 70% of the styles three days after pollination (Figure 2B). At this time a mean number of eight pollen tubes per style reached the hypanthium entrance (Figure 2C). This number was maintained until the fifth day after pollination, when for unknown reasons, a second wave of pollen tubes arrived. At seven days after pollination, there was a mean of 20 pollen tubes at the hypanthium entrance.

**Figure 3:**
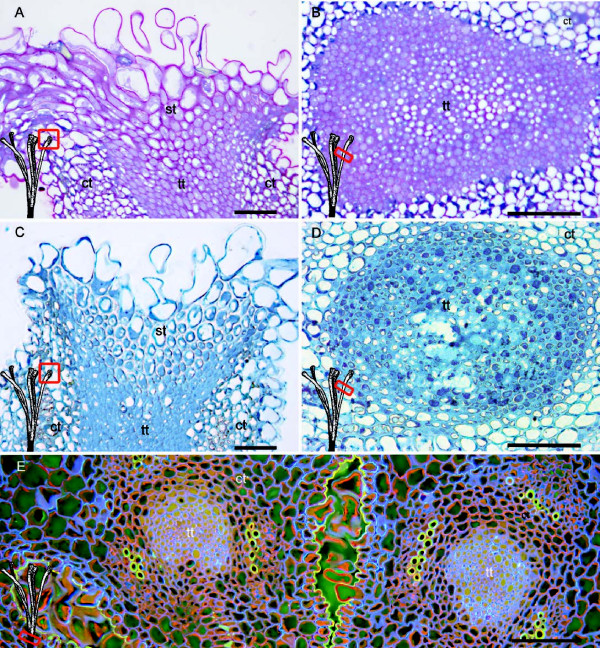
**Stigma and stylar transmitting tissue of *Malus *x *domestica *at anthesis. (A)** Stigma and stigmatoid tissue with intercellular spaces filled with insoluble polysaccharides after staining with Periodic acid Shiffs-PAS reagent counterstained with toluidine blue. **(B)** The extracellular matrix of the transmitting tissue also stained for polysaccharides. **(C)** Absence of general protein staining with Naphtol Blue Black in the intercelullar space of the stigmatoid tissue, contrasting with **(D)** strong reaction both within cells and in the intercellular space of the style transmitting tissue with the same staining. **(E)** Reduced area of the transmitting tissues at the stylar base in the multicarpelar apple pistil stained with acridine orange counterstained with aniline blue. Light micrographs of 2 μm longitudinal stigmatic sections **(A,C)**, and transversal style sections in the upper style (**B,D** - red squares), and at the stylar base (**E**-red square). Cortical tissue (ct), stigmatoid tissue (st), transmitting tissue (tt). Scale bars: 50 μm.

At anthesis, absence of starch grains contrasted with the presence of insoluble polysaccharides in the intercellular spaces of both the stigmatoid tissue and the upper stylar transmitting tissue (Figure 3A, B). However, staining for proteins with Naphtol Blue Black gave no reaction in the intercellular spaces of the stigmatoid tissue (Figure 3C) in contrast to the strong protein staining both within and between the cells of the transmitting tissue (Figure 3D). Since a reduction in the population of the growing pollen tubes was observed and only 8% of the germinated pollen grains on the stigma reached the stylar base, we searched for a possible reduction in the area occupied by the transmitting tissue at the style base. While in the upper style the transmitting tissue cross section area was 14.900 μm^2^, at the bottom of the style (Figure 3E) it was reduced to 2.500 μm^2^, a six fold reduction.

### Polysaccharides in the style

**Figure 4:**
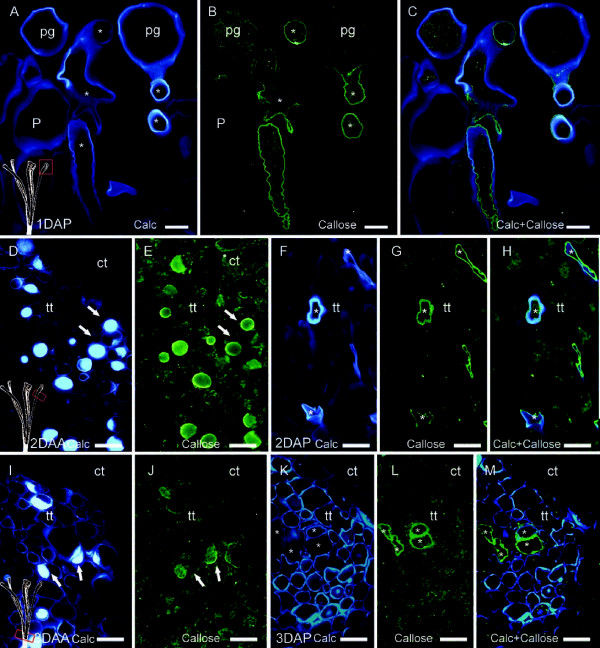
**Calcofluor staining and callose inmunolocalisation in unpollinated and pollinated pistils of *Malus *x *domestica *at the stigma (A-C), mid- style (D-H), and at the style base (I-M). (A)** Cellulose stained pollen grain intine, pollen tube walls, and stigmatoid tissue cell walls one day after pollination (1DAP). **(B)** Same section immunolocalising linear β-(1,3)-glucans (callose) along the inner side of the pollen tube wall, **(C)** overlapped images of both cellulose and callose. **(D)** Cellulose and other polysaccharides detected (arrows) in the cells of the style transmitting tissue of unpollinated pistils two days after anthesis (2DAA). **(E)** Callose immunolocalization (fluorescent green-arrows) in an unpollinated pistil (2DAA). **(F)** Pollen tubes (asterisks) in the transmitting tissue two days after pollination (2DAP). **(G)** Callose immunolocalized at pollen tube wall, but not in the transmitting tissue cells of pollinated pistils. **(H)** Overlapped image of callose and cellulose in the pollen tube wall two days after pollination. **(I)** Cellulose and other polysaccharides at the base of the stylar transmitting tissue (arrows) in unpollinated pistils accumulated later, by three days after anthesis (3DAA). **(J)** Callose co-localized with cellulose (arrows) in the transmitting tissue of unpollianted pistils. **(K)** While cellulose and other polysaccharides were no longer detected in pollinated pistils three days after pollination (3DAP), **(L)** pollen tube walls labelled for callose (asterisks) in cross sections. **(M)** Overlapped image of calcofluor white and callose at the stylar base. Calcofluor staining **(A, D, F, I, K)**, linear β-(1–3)-glucans immunolocalization with FITC secondary Ab labelling in fluorescent green **(B, E, G, J, L)**, and merged images callose-calcofluor **(C, H, M)** in 4μm longitudinal **(A-C)**, and transversal **(D-M)** sections of upper **(A-C)**, middle **(D-H)**, and lower **(I-M)** style. Asterisks indicate pollen tubes. ct, cortical tissue; P, papillae; pg, pollen grain; tt, stylar transmitting tissue. Scale bars: 10 μm.

Since pollen tube walls are composed of pectins, cellulose and callose [40], unpollinated and pollinated styles were stained with calcofluor white to visualize cellulose and other polysaccharides [48], and immmunolabelled for the linear β-(1–3) glucans, the backbone of callose [49]. After pollination, the papillae cell walls in the stigma stained for cellulose, but were devoid of callose (Figure 4A, B). Germinating pollen grains, on the other hand, displayed callose localization in the pollen tube wall (Figure 4C).

In the style, unpollinated flowers were positive for cellulose and other polysaccharides, which accumulated in a centripetal fashion from the edges to the centre in the transmitting tissue cells (Figure 4D). In addition, immunolocalization revealed a callose layer surrounding those saccharidic accumules (Figure 4E). However, in pollinated styles, callose was only detected in the pollen tube walls (Figure 4F, G), but not in transmitting tissue cells (Figure 4H). At three days after anthesis, unpollinated styles accumulated carbohydrates following a basipetal fashion and were present at the base of the style (Figure 4I, J). In pollinated styles, cellulose (Figure 4K) and callose (Figure 4L) were present in pollen tube walls, but were not detected in the transmitting tissue cells (Figure 4K, L and M).

### Different glycoprotein epitopes in the style

**Figure 5:**
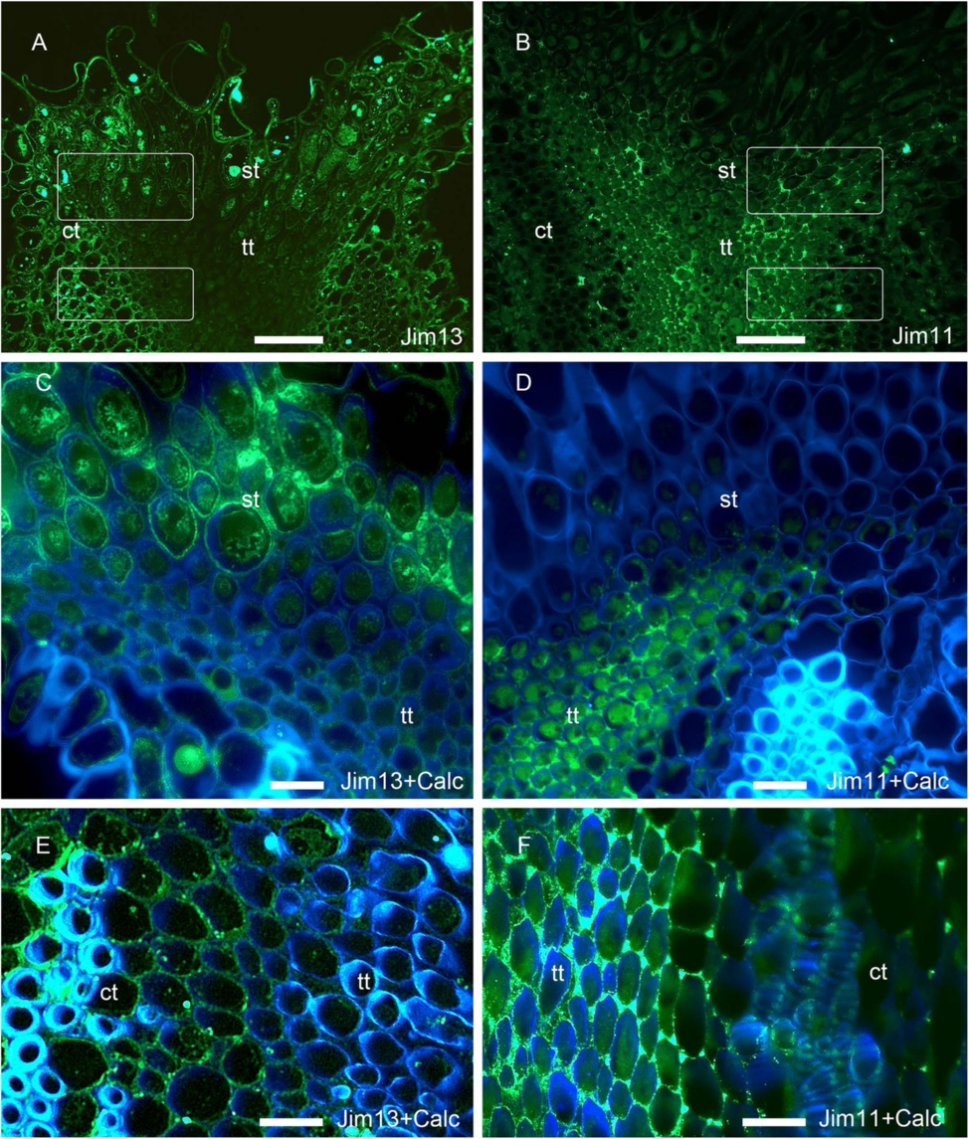
**Glycoproteins in the stigmatoid and stylar transmitting tissue of *Malus *x *domestica, *at anthesis*. *(A)** Arabinogalactan proteins labelled with JIM13 mAb were present in the cortical and stigmatoid tissues, but were absent in the stylar transmitting tissue. **(B)** In contrast, extensin epitopes were conspicous in the transmitting tissue of the style. **(C)** While the stigmatoid tissue intercellular space positively reacted for the presence of arabinogalactan proteins epitopes recongized by JIM13 mAb, **(D)** intercellular spaces of the contiguous transmitting tissue was marked by the presence of extensins. **(E)** The upper style further lacked JIM13 arabinogalactan protein epitopes in the transmitting tissue. **(F)** In contrast the intercellular space in the style transmitting tissue contained extensins. Longitudinal 4μm sections of the stigma-style transition **(A-D)**, and the style **(E-F)**, tagged either for arabinogalactan proteins with JIM13 mAb **(A,C,E)**, or extensins with JIM11 mAb **(B,D,F)**. Merged images of FITC labelling (green) with calcofluor white (blue) **(C-F)**. White squares note the location of the magnified **C,E** and **D,F** pictures in each column respectively. ct, cortical tissue; st, stigmatoid tissue; tt, stylar transmitting tissue. **A-B** scale bars: 50 μm; **C-F** scale bars: 10 μm.

At anthesis, arabinogalactan proteins detected with JIM13 mAb, were observed in the cortical tissue and the stigmatoid tissue (Figure 5A and [45]). However, they were lacking in the transmitting tissue of the style (Figure 5A). In contrast, extensin epitopes recognised by JIM11 mAb were lacking in the stigmatoid tissue, but were conspicuously detected in the extracellular spaces of the style transmitting tissue (Figure 5B). A closer view to the stigma showed that while AGPs recognized by JIM13 mAb filled the intercellular spaces of the stigmatoid tissue (Figure 5C), extensins recognized by JIM11 mAb filled the intercellular space of the contiguous tissue, the transmitting tissue of the style (Figure 5D). This was further confirmed along the style, and while AGP epitopes were detected in the cell walls of the cortical tissue (Figure 5E), they were not in the stylar transmitting tissue. On the other hand, and consistent with observations made closer to the stigma, the intercellular matrix of the transmitting tissue showed a strong presence of extensin epitopes (Figure 5F).

**Figure 6:**
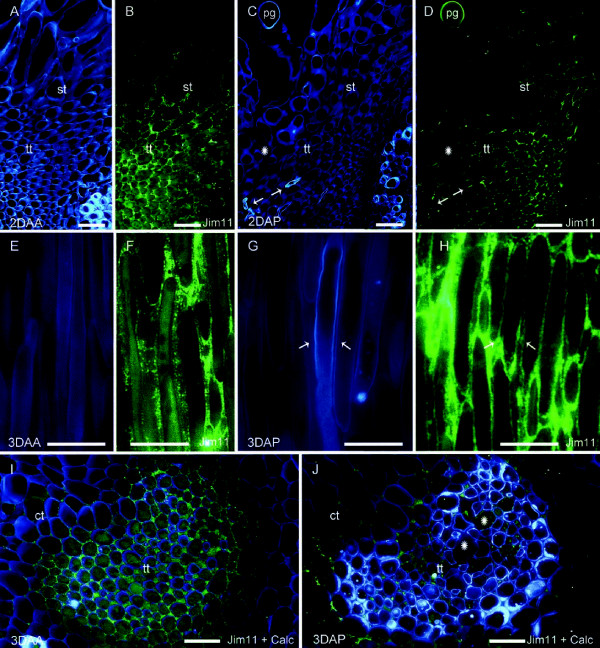
**Extensins in pistils of *Malus *x *domestica, *unpollinated (A,B,E,F,I) and pollinated (C,D,G,H,J) flowers; at the stigma (A-D), middle of the style (E-H), and the style base (I-J). (A)** Cell walls of the transmitting tissue stained for cellulose, and **(B)** intercellular spaces immunolocalized for extensin epitopes (fluorescent green) in the upper stylar transmitting tissue two days after anthesis (2DAA). **(C)** Transmitting tissue stained cell walls and pollen tubes (arrows) two days after pollination (2DAP). **(D)** Extensin epitopes decreased detectability in the extracellular spaces, particularly in the areas traversed by the pollen tubes (stars). **(E)** Cells of the transmitting tissue in an unpollinated style three days after anthesis (3DAA). **(F)** Same section showed extensin epitopes recognized by JIM11 mAb in the extracellular domains of the dense cytoplasmic elongated cells. **(G)** Pollen tube (arrows) traversing the extracelluar matrix of the transmitting tissue three days after pollination (3DAP). **(H)** Same section immunolocalized for JIM11 extensin epitopes in the extracellular matrix showing conspicuous contact with pollen tube walls. **(I)** Overlapped images of calcofluor and JIM11 extensin epitopes; extensins fill intercellular spaces at the style base three days after anthesis (3DAA). **(G)** Same area at the style base devoid of extensins (asterisks) following pollen tubes passage. Style 4 μm longitudinal **(A-H)** or transversal **(I-J)** sections stained with Calcofluor white **(A, C, E, G)**, immunolocalized for extensin epitopes showing FITC labelling in fluorescent green **(B, D, F, H)**, and merged images of calcofluor white and FITC signalling **(I-J)**. ct, cortical tissue; pg, pollen grain; st, stigmatoid tissue; tt, transmitting tissue. **(A-D)** scale bars: 50μm; **(E-J)** scale bars: 10 μm.

Due to their location at places of pollen tube elongation, a possible role of extensins in pollen tube growth was evaluated by the comparison of pollinated and unpollinated styles. In unpollinated flowers, the intercellular spaces of the upper part of the transmitting tissue (Figure 6A) strongly reacted to extensin epitopes and accumulated to higher levels following anthesis (Figure 6B). In pollinated flowers, pollen tubes were observed in the intercellular spaces (Figure 6C); extensin epitopes were detectable in the transmitting tissue, however, the accumulation of extensins was at far lower levels compared to unpollinated flowers (Figure 6D). In the middle of the style, three days after anthesis cellulosic walls (Figure 6E) delimited the dense cytoplasmic large cells of the transmitting tissue, as well as extensins conspicuously filled the intercellular spaces (Figure 6F). In pollinated flowers, as pollen tubes elongated along the intercellular domains of the style transmitting tissue (Figure 6G), a constant contact between pollen tube walls and an extensin-rich extracellular matrix was observed (Figure 6H). A similar pattern could be seen at the style base, where the extracellular matrix of the transmitting tissue in unpollinated pistils conspicuously showed extensin epitopes (Figure 6I). Interestingly, extensin epitopes were detected at much lower levels in pollinated flowers, particularly in areas closer to pollen tube passage (Figure 6J).

Taking together these observations, show that β-glucan polysaccharides and extensins developmentally accumulated in a centripetal and basipetal way all along the stylar transmitting tissue, but these compounds could no longer be detected following pollen tube passage.

## Discussion

Results in this work suggest that extensins and polysaccharides of the style transmitting tissue contribute to the pollen tube growth in the apple style. While they accumulate in unpollinated styles, they can no longer be detected in pollinated styles. These extensins and polysaccharides appear only in the style, not in the stigma, strongly suggesting that they contribute to the growth of pollen tubes in the style.

### The change from autotrophic to heterotrophic pollen tube growth

Reports in a wide range of species with different style types have provided evidence for a nutritive role for the transmitting tract, where starch depletion was coincident with pollen tube elongation [9,16,50-53]. The immunocytological localization described in this work provides further support for this idea. While starch was not detected in the apple transmitting tissue, polysaccharides accumulated in the extracellular matrix of unpollinated styles. Polysaccharide composition and presence was determined by staining with PAS for general insoluble polysaccharides, calcofluor for cellulose and pectins detection, and by immunolocalization of linear β-(1,3)-glucan segments, the backbone of callose.

Callose has been repeatedly detected in the inner wall of the pollen tube shank in different angiosperms [16,38,54]. It has been suggested that its function is related to a resistance to pressure with some degree of permeability [55]. Those functional capacities are also supported by the pecto-cellulosic composition of the pollen tube wall [44,56]. At the pollen tube extensible tip, exocytosis of *de novo* materials appears to be the main contributor to pollen tube elongation, but also endocytosis is required to recycle and regulate wall materials, such as membrane proteins [57]. Even though callose is primarily produced by the internal machinery of the pollen tubes, the incredibly high amounts of this polysaccharide in pollen tubes could also result from the intake of precursors from the pistil tissues. There is scarce evidence, however, regarding the provenance of the callose and cellulose required to build the pollen tube wall [44]. While stigma cells were devoid of callose, germinating pollen grains were rich in this β-glucan, with a strong localization signal at the inner side of the growing pollen tube walls after pollen germination. Whereas in unpollinated styles these and other polysaccharides accumulated in the transmitting tract, they were no longer detected in pollinated pistils. Callose accumulation in unpollinated styles could play a part in defence, but the disappearance of these β-glucans in pollinated pistils strongly suggests a role for maternal support of pollen tube growth in the style [8,16,17].

### Strategies in the style controlling pollen tube growth

The observed reduction in the transmitting tissue area along the style, together with a reduction in the β-glucans and extensins contributing to pollen tube elongation suggest that the style could play a role in pollen tube competition. A reduction in the area of the style that pollen tubes traverse has been reported in species with both solid [58,59], and hollow styles [53]. It was proposed that physical constraints due to the funnel-like structure of the style were one of the main factors favouring pollen tube competition and selection along the style, resulting in higher quality off-spring produced [37,60-62]. Results from this work support this model in which a reduction in the area accumulating provisions limited the available resources and resulted in only a small percentage of the pollen tubes reaching the base of the style. However, it is not yet clear whether the first pollen tubes that reach the base of the style are more successful at achieving fertilization [63]. Instead of arriving all at once, apple pollen tubes arrive at the stylar base in two waves, enabling selection within the style first, followed by a second selection at the ovary site. All together data herein suggest a finely-tuned system of maternal and paternal interactions with implications on the sexual conflict in plants [64], and thus sporophytic female selection of male gametophytes might be influenced by specific spatial arrangement of glycoproteins in the maternal transmitting tissue, the pathway of pollen tubes.

### Different glycoprotein epitopes for different pistil territories

While the results suggest that the style might favours pollen tube competition, the question remains as to why the pollen tube growth rate is slower in the stigma than in the style. This may be related to the different glycoprotein epitopes present in the different territories of the pollen tube pathway. Within the apple pistil, arabinogalactan proteins were abundant in the stigmatoid tissue, but were lacking in the stylar transmitting tissue. Strikingly, extensins were not detectable in the stigma with the antibodies used, but were present at abundant levels in the intercellular spaces of the style transmitting tissue. This specialized glycoproteins distribution is surprising given that the stigma and the style are contiguous tissues. In Solanaceae pistils, extensin-like proteins (PELPIII) have been reported to play a part in interspecific incompatibility through the initial localisation in the stylar transmitting tract followed by relocation to the inner callose layer of growing pollen tubes [33-35]. Arabinogalactan proteins have also been localised in the transmitting tissue of different angiosperm species ranging from early-divergent angiosperms [65] to eudicots such as *Nicotiana*, where a glycosylation gradient related to pollen tube nutrition has been reported [28-31]. Furthermore, expansins in the extracellular matrix have been associated with relaxation of the intercellular space for pollen tube penetration in both the stigma and the style in grasses [66-69]. The stigma-style transition is also marked by an acceleration of pollen tube growth rate. This increase in the rate of growth was previously reported in other species including *Petunia*[15], peach [9], kiwifruit [50], and apricot [70]. Pollen tube growth in the style also appears to be faster than in the ovary [11,71], and these differences have been related to the requirement for maturation of ovary structures. The acceleration of pollen tube growth rate in the style could be due to developmental events within the pollen tube itself, such as the deposition of the first callose plug, the completion of mitosis II, or by an internally programmed shift to heterotrophic growth. Results herein suggest that the different composition of glycoproteins observed in the stigma and the style could also play a part.

Our results show that extensins also have a role in the apple stylar transmitting tract. Their undetectability following the passage of pollen tubes provides additional suggestion of heterotrophic pollen tube growth in the style. Extensins are essential builders of new plant cell walls [72], and the close relationship with growing pollen tubes along the pistil suggests a role for extensins in pollen tube wall building and acceleration of the growth rate in the style. Extensins have been shown to play a biochemical role in the tensile strength of growing cells [73]. However, given the presence of a leucine rich repeat (LRR) domain in some extensins [34], a role as recognition-signal molecules cannot be ruled out. Among the best characterised, LRR chimera extensins (Pex) in maize and tomato pollen tubes have been suggested to play a role in pollen-style interactions [37,38,73]. This work shows that in the apple flower pollen tube growth is modulated by the different pistil tissues it has to traverse. Further work will elucidate whether this is a conserved pattern in other angiosperms.

## Conclusions

Taken together, these results strongly suggest that precise glycoprotein distribution in the different pistil tissues is important in the finely-tuned pollen-pistil interaction. The switch, from an autotrophic pollen tube growth in the stigma to heterotrophic growth in the style, occurs with a two-fold acceleration in pollen tube growth rate. This is accompanied by the presence of extensins and β-glucans which accumulate in a basipetal fashion in the style, but not in the stigma. While these resources are present in unpollinated flowers, they were no longer detectable in pollinated flowers when the pollen tubes passed by, suggesting a contribution to rapid wall building in pollen tubes. A reduction in the number of pollen tubes reaching the base of the style is accompanied by a basipetal reduction, both in the area and in the resources available along the style, strongly suggesting that this reduction favours pollen tube competition. This observations open a door for understanding species-specific pollen-pistil interactions and for the possible role these interactions have in prezygotic mechanisms of speciation.

## Methods

### Plant material

Flowers from apple trees cv Golden Delicious Spur grown in the province of Huesca (Spain), 461m above sea level were used in this study. To evaluate pollen tube elongation along the style, a bunch of 120 flowers were emasculated at balloon stage and left for 24 h until hand pollination [74].

Since apples are self-incompatible, anthers from the compatible cv Royal Gala were collected from flowers at an advanced balloon stage and left to dry on paper at room temperature around 20ºC for 24–48 h until dehiscence. Pollen was sieved with a mesh with a diameter pore of 0.26 mm and then stored at -20ºC until required. Half of the flowers -60- were left without pollination, and the other half -60- were hand pollinated with a paint brush.

### Pollen tube growth rate evaluation along the style

To monitor pollen tube growth rate in the stigma and the style, following hand pollinations, five flowers -30 gynoecia- were sampled at intervals of two hours up to twelve hours, and later the same number of flowers was daily sampled and fixed in FAA (formalin: acetic acid: 70% ethanol) (1:1:18) [75] for seven days after pollination. After being fixed, the pistils were washed three times in distilled water for one hour each, and left in 5% sodium sulphite for 24 h. Samples were autoclaved for 10 min at 1 Kg cm^-2^ in 5% sodium sulphite, and then the individual styles were squashed onto glass slides with 0.1% aniline blue in 0.1NK_3_PO_4_ to visualize callose [76,77] and pollen tubes [41]. Pollen tubes were visualized with a LEICA DM2500 fluorescence microscope bearing a 340/400 nm filter, and pollen tube number was counted at the hypanthium entrance. Percentages of styles with pollen tubes at the stylar base were recorded in pollinated pistils fixed at different days after pollination, and were sequentially compared by days after pollination using chi-square homogeneity test at a *P*?≤?0.05. Both percentages of styles traversed and mean number of pollen tubes at the stylar base were subjected to mean comparison by one way ANOVA, and significant independent groups were separated by Duncan multiple range test at a *P*?≤?0.05. Statistical analysis was performed with the SPSS software (SPSS Inc., Chicago, USA).

### Histochemical preparations

Flowers for histochemical examination were selected according to the pollen tube kinetics results, at anthesis, two, and three days after pollination. Two flowers - 10 styles - per day were fixed in 2.5% glutaraldehyde in 0.03M saline phosphate buffer pH7.3 for 4 h [78]. Then the pistils were washed in 0.03M saline phosphate buffer and sequentially dehydrated in an ethanol series (30%, 50%, 70%, and 96%), leaving them one hour in each ethanol concentration. The gynoecia were left for five days in the embedding solution at 4ºC, and then embedded in JB4 plastic resin (Polysciences Inc., 0226A). Both longitudinal and transversal sections 2μm thick were cut on a LEICA EM UC6 ultramicrotome with a glass knife and then placed onto distilled water on a glass slide previously coated with 1% gelatine. Polysaccharides were stained with periodic acid shift reagent-PAS [79] counterstained with 0.02% Toluidine Blue for general structure, and proteins with 0.25% Naphtol Blue Black in 1% acetic acid [80]. Also 0.07% calcofluor white for cellulose [48] and other polysaccharides [52], 0.01% auramine in 0.05M phosphate buffer for cutin and lipids [81], and 0.01% acridine orange in 0.03% phosphate buffer, pH7.4 [82] were used to observe the stylar morphology.

Slides were observed under bright field LEICA DM2500 microscope carrying 100W light source, and photographs were obtained with a Leica DFC320 camera linked to the software Leica Application Suite. Fluorescence observations were done with the same microscope provided with an epifluorescence source and connected to a CANON Power Shot S50 camera linked to the CANON Remote Capture software. Filters used were 355/455 nm for calcofluor white, and 450/510 nm for auramine and acridine orange stained sections.

### Immunolocalization of callose, AGPs and extensins

Two flowers per day from anthesis, two and three days after pollination were fixed in 4% formaldehyde freshly prepared from paraformaldehyde in 1x phosphate saline buffer (PBS) pH7.3, left overnight at 4ºC, and conserved then at 0.1% formaldehyde solution [83]. Then the pistils were dehydrated in an acetone series (30%, 50%, 70%, 90%, 100%), and embedded in Technovit 8100 (Kulzer and Co, Germany) for two days. The resin was polymerized at 4ºC, and sectioned at 4 μm thickness. Sections were placed in a drop of water on a slide covered with 2% (3-Aminopropyl) triethoxysilane - APTEX (Sigma-Aldrich), and dried at room temperature. Callose was identified with the anticallose antibody (AntiCal) that recognises linear β-(1,3)-glucan segments (anti-β-(1,3)-glucan; immunoglobulin G1), Biosupplies, Australia [49]. As a secondary antibody, Alexa 488 fluorescein isothiocyanate (FITC)-conjugated anti-mouse IgG was used (F-1763; Sigma). Additionally, a monoclonal antibody (mAbs) JIM13 [84] against AGPs glycosyl epitopes, and one mAb JIM11 [85] against extensin epitopes were obtained from Carbosource Services (University of Georgia, USA). Secondary antibodies were anti-rat IgG conjugated with the same Alexa 488 used above. Sections were incubated for 5 min in PBS pH7.3 followed by 5% bovine serum albumin (BSA) in PBS for 5 min. Then, sections were incubated at room temperature for 1h with AntiCal primary mAb, JIM13, and JIM11. After that, three washes in PBS of 5 minutes each preceded the incubation for 45 min in the dark with a 1/25 diluted secondary fluorescein isothiocyanate (FITC) conjugated with the antibody in 1% BSA in PBS, followed by three washes in PBS [83]. Sections were counterstained with calcofluor white for cellulose [86], mounted in PBS or Mowiol, and examined under a LEICA DM2500 epifluorescence microscope connected to a LEICA DFC320 camera. Filters were 355/455 nm for calcofluor white and 470/525 nm for the Alexa 488 fluorescein label of the antibodies (White Level?=?255; Black Level = 0; ϒ?=?1). Exposur (Exp) times were adapted to the best compromise in overlapping photographs for each antibody: AntiCal, Exp.?=?15.30ms (Calcofluor Exp. = 1.20ms); JIM13 Exp.?=?2.52ms (Calcofluor?=?0.41ms); JIM11, Exp. = 31.59 ms (Calcofluor Exp. = 1.40ms). Brightness and contrasts were adjusted to obtain the sharpest images with the Leica Application Suite software.

## Abbreviations

Exp: Exposure times; GABA: γ-aminobutiric acid; HPRGs: Hydroxyproline-rich glycoproteins; AGPs: Arabinogalactan-proteins; AntiCal: anti-β-(1,3)-glucan monoclonal antibody; TTS: Transmitting tissue specific glycoprotein of *Nicotiana tabacum*; EXTs: Extensins; PELPIII: Solanaceae pistil specific extensin-like protein; Pex: Pollen specific extensin-like proteins; LRR: Leucine rich repeat domain; FAA: Formalin - acetic acid - alcohol; PBS: Phosphate buffered saline; mAb: Monoclonal antibody; FITC: Fluorescein-isothiocyanate.

## Competing interests

Both authors declare no competing interests.

## Authors’ contributions

MH designed and supervised the work. JML performed the experimental work. Both authors participated in the data analysis, discussion of results, and writing the manuscript. Both authors read and approved the final manuscript.
